# Carnitine O-octanoyltransferase (CROT) deficiency in mice leads to an increase of omega-3 fatty acids

**DOI:** 10.3389/fmolb.2024.1374316

**Published:** 2024-07-15

**Authors:** Takehito Okui, Shiori Kuraoka, Masaya Iwashita, Rei Itagawa, Taku Kasai, Masanori Aikawa, Sasha A. Singh, Elena Aikawa

**Affiliations:** ^1^ Center for Interdisciplinary Cardiovascular Sciences, Division of Cardiovascular Medicine, Department of Medicine, Brigham and Women’s Hospital and Harvard Medical School, Boston, MA, United States; ^2^ Center for Excellence in Vascular Biology, Division of Cardiovascular Medicine, Department of Medicine, Brigham and Women’s Hospital and Harvard Medical School, Boston, MA, United States; ^3^ Channing Division of Network Medicine, Department of Medicine, Brigham and Women’s Hospital and Harvard Medical School, Boston, MA, United States

**Keywords:** CROT, metabolomics, acyl-carnitine, knockout mouse, cardiovascular calcification

## Abstract

**Introduction:** Carnitine O-octanoyltransferase (CROT) is a well-established peroxisomal enzyme involved in liver fatty acid oxidation, but less is known about its recently discovered role in promoting vascular calcification, and whether CROT-dependent liver metabolism contributes to the latter. To date, CROT function in the context of calcification potential has been conducted in the dyslipidemic low-density lipoprotein receptor-deficient (*Ldlr−/−*) mice.

**Objectives:** To differentiate peroxisome and CROT-dependent lipid biology from that of lipoprotein-mediated lipid biology, we therefore conducted a metabolomic analysis of the liver and plasma of normolipidemic CROT-deficient (*Crot−/−*) mice.

**Methods:** We performed LC-MS-based metabolomics on liver and plasma derived from *Crot−/−* and *Crot* +/− mice and sibling *Crot+/+* mice, using a dual-phase metabolite extraction protocol, and multiple LC-MS acquisition strategies.

**Results:** We identified between 79 to 453 annotated metabolites from annotated metabolites from liver samples, and 117 to 424 annotated metabolites from plasma samples. Through differential abundance analysis, we determined that omega-3 fatty acids such as EPA, DPA, and DHA were higher in the liver of *Crot−/−* and *Crot* +/− mice than *Crot+/+* mice. EPA were higher in plasma of *Crot−/−* mice than *Crot+/+* mice. We also determined that the anti-inflammatory dicarboxylic acids, tetradecanedioic acid and azelaic acid, were higher in the plasma of CROT-deficient mice.

**Conclusion:** Our study associated genetic CROT deletion with increased levels of anti-inflammatory molecules in mouse liver and plasma. These results suggest a potential mechanism for anti-calcification effects of CROT suppression and the potential use of omega-3 fatty acids as biomarkers for future CROT inhibition therapies.

## 1 Introduction

Various metabolic pathways involve the peroxisome, including phospholipid synthesis, isoprenoid biosynthesis, fatty acid α-oxidation, and notably the shortening of fatty acids by oxidation of their β carbon also known as fatty acid β-oxidation (Kunau et al., 1995). Fatty acid β-oxidation occurs in the peroxisomes of animals, plants and fungi, but also in the mitochondria of animals (Kunau et al., 1995). This oxidation pathway begins with the conjugation of a free fatty acid to coenzyme A (CoASH) for subsequent cycling through the four steps of β-oxidation, releasing acetyl-CoA each cycle until the entire fatty acid chain is broken down (Kunau et al., 1995). Peroxisomes and mitochondria accept distinct classes of fatty acids. For instance, peroxisomes accept very long chain, polyunsaturated, branched, and sterol derivatized fatty acids whereas mitochondria accept primarily medium- and short-chained fatty acids (Lazarow and De Duve, 1976; Hiltunen et al., 1986). Another main distinction between the two organelles is that peroxisomes catalyze only partial fatty acid β-oxidation; that is, very long-chained fatty acids undergo oxidation for only a few cycles until they are shortened to medium-chained fatty acyl-CoA derivatives such as octanoyl-CoA (Lazarow and De Duve, 1976; Hiltunen et al., 1986). In order for these partially oxidized fatty acids to be fully broken down, they are transferred to the mitochondria, but as acyl-carnitines since mitochondria are impermeable to acyl-CoAs (Jakobs and Wanders, 1995). Carnitine O-octanoyltransferase (CROT) is a peroxisomal enzyme that catalyzes the reversible reaction of L-carnitine and acyl-CoA to acyl-L-carnitine and CoASH, permitting the peroxisome-generated medium-chained fatty acids to be substrates for mitochondria.

Our recent proteomics-based investigation to identify proteins and mechanisms that promote vascular calcification revealed that CROT is induced when human smooth muscle cells undergo osteogenic differentiation (Okui et al., 2021). Specifically, pathway analysis of proteins whose abundances increased in the osteogenic condition yielded multiple pathways relating to peroxisomes and lipid metabolism that ultimately pointed to CROT for further interest. SiRNA silencing of CROT in smooth muscle cells inhibited their calcification potential but also increased the omega-3 polyunsaturated fatty acid, eicosapentaenoic acid [EPA, 20:5 (n-3)] (Okui et al., 2021). Furthermore, in low density lipoprotein receptor-deficient mice (*Ldlr−/−* mice), a model for atherosclerosis that also develops vascular calcification, systemic genetic deletion of CROT reduced calcification in the carotid artery and aortic arch independent of circulating lipid profile, and with no impact on normal bone development (Okui et al., 2021).

Peroxisome-dependent fatty acid beta-oxidation and CROT have been studied primarily in the context of liver metabolism, but also using fibroblasts derived from patients lacking mitochondrial fatty acid transfer proteins and the epithelial-like hepatocellular carcinoma cell line, HepG2 (Bronfman et al., 1979; Jakobs and Wanders, 1995; Kunau et al., 1995; Le Borgne et al., 2011). In HepG2 cells, overexpression of CROT decreased medium-to long-chained fatty acids and very long-chained saturated or monounsaturated fatty acids; whereas the opposite resulted from siRNA-mediated reduction of CROT (Le Borgne et al., 2011). However, unlike the observed increase in EPA in smooth muscle cells treated CROT siRNA (Okui et al., 2021), polyunsaturated fatty acids did not change with CROT deficiency in HepG2 cells (Le Borgne et al., 2011). Lipid metabolism, CROT activity, and peroxisomal functions are context dependent across organisms, and across tissues and cell types within an organism (Kunau et al., 1995), thus these disparate findings are not unexpected.

To better understand the physiological function of CROT in mice, and in a genetic background without complications due to dyslipidemia (*Ldlr−/−*), we conducted a metabolome analysis of livers isolated from CROT-deficient mice in a wildtype (C57BL/6J) background.

## 2 Materials and methods

Additional methods are included in the Supplementary material.

### 2.1 CROT-deficient mice

The CRISPR-Cas9 method (The Jackson Laboratory) was used to delete exon three that includes the start codon in *Crot* in C57BL/6J mice (Okui et al., 2021). Specifically, single-guide RNA targeting intron 2 (5′-ACA​CCA​CAC​TAC​CTG​GGG​TT-3′, 5′-AAC​ACC​ACA​CTA​CCT​GGG​GT-3′, 5′-CTC​TAA​CAC​CAC​ACT​ACC​TG-3′, 5′-CCA​CCC​AAC​CCC​AGG​TAG​TG-3′) and intron 3 (5′-CCT​TGT​ACT​AAG​TCC​ACG​GA-3′, 5′-TCC​TTG​TAC​TAA​GTC​CAC​GG-3′, 5′-ATC​TCC​TTG​TAC​TAA​GTC​CA-3′, 5′-CCC​TCC​GTG​GAC​TTA​GTA​CA-3′) of *Crot* were used. Male mice were used in this initial study characterizing CROT deficiency due to known sex differences in fatty acid and lipid metabolism (Palmisano et al., 2018) that could impact data interpretation.

### 2.2 Blood sampling

10-week-old wild-type (*Crot+/+*), *Crot* heterozygous (*Crot+/−*) and homozygous (*Crot−/−*) male mice were anesthetized with pentobarbital, and 0.7 mL blood was drawn from inferior vena cava into microtubes with EDTA-2Na (final concentration was 1.4 mg/mL in blood) and centrifuged at 2,000 × *g* for 15 min at 4°C. Plasma aliquots of 100 µL were stored at −80°C.

### 2.3 Liver sample preparation for metabolomic analysis

All solvents were purchased from Fisher Scientific and are LC-MS grade, unless otherwise stated. The slightly modified metabolite extraction workflow established previously (Supplementary Figure S1) (Ferrarini et al., 2019). We analyzed liver from n = 5 mice per genotype (*Crot+/+, Crot+/−*, and *Crot−/−*). We added 1 mL of prechilled 50% methanol to 10 mg of liver samples and sonicated them. After centrifuging samples at 14,000 × *g* for 15 min at 4°C, samples were incubated on ice for an additional 20 min and centrifuged at 14,000 × *g* for 20 min at 4°C. 400 μL of supernatant was transferred to new microfuge tubes (pellets were kept as P1), to which we added 400 µL of prechilled acetonitrile, and then vortexed. After incubating samples for another 20 min on ice and centrifuging at 14,000 × *g* for 15 min at 4°C, we transferred the supernatants to new microfuge tubes (pellets were kept as P2) for subsequent evaporation using a vacuum evaporator at room temperature. The evaporated pellets were resuspended with 125 µL of reagent A (25% acetonitrile, 50% methanol and 25% water).

For the pellet fractions (P1 and P2), we added 500 and 125 µL of reagent B (75% dichloromethane (Fisher Scientific, AA22917K2 HPLC-grade) and 25% methanol), respectively. After sonicating each pellet on ice, we combined P1 and P2 sonicates and centrifuged for 20 min at 14,000 × *g* for 15 min at 4°C. 600 μL of supernatant was transferred to new microfuge tubes to which we added 600 µL of prechilled acetonitrile, and then vortexed. After incubating samples for 20 min on ice and centrifuging at 14,000 × *g* for 15 min at 4°C, we transferred the supernatants to new microfuge tubes for subsequent evaporation using a vacuum evaporator at room temperature. The evaporated pellets were resuspended with 125 µL of reagent A. Any residual precipitate was cleared by centrifuging at 14,000 × *g* for 10 min at 4°C. The cleared samples were transferred to autosampler vials (9 mm silicone vials/screw thread caps, Fisher Scientific C5000-54B), and immediately analyzed using LC-MS/MS. In addition, we prepared quality control (QC) samples by pooling each genotype sample for QC-group and all samples for QC-all.

### 2.4 Plasma sample preparation for metabolomic analysis

All solvents were purchased from Fisher Scientific and are LC-MS grade, unless otherwise stated. The slightly modified metabolite extraction workflow established previously (Supplementary Figure S1) (Ferrarini et al., 2019). We analyzed plasma from n = 5 mice per genotype (*Crot+/+, Crot+/−*, *Crot−/−*). We added 1 mL of reagent B to 100 µL of mouse plasma or water (blank, mock control) and vortexed. After sonicating samples using an iced water bath sonicator for 2 min, samples were incubated on ice for an additional 20 min and centrifuged at 14,000 × *g* for 20 min at 4°C. The upper and lower fractions were transferred to separate new microfuge tubes, to which we added 300 μL and 800 µL of prechilled acetonitrile, respectively, and then vortexed. After incubating samples for another 20 min on ice and centrifuging at 14,000 × *g* for 15 min at 4°C, we transferred the supernatants to new microfuge tubes for subsequent evaporation using a vacuum evaporator at room temperature. The evaporated pellets were resuspended with 80 µL of reagent C (0.1% formic acid, 50% methanol and 49.9% water) and 80 µL reagent A, respectively. Any residual precipitate was cleared by centrifuging at 14,000 × *g* for 10 min at 4°C. The cleared samples were transferred to autosampler vials, and immediately analyzed using LC-MS/MS. In addition, we prepared QC samples by pooling each genotype sample for QC-group and all samples for QC-all.

### 2.5 Mass spectrometry for global metabolite profiling

*Injection queue*—A total of 30 unique liver and plasma metabolite samples were analyzed for each extraction method: n = 3 *Crot* genotypes x five mice plasma samples x two metabolite extract replicates for each upper and lower fraction; and n = 3 *Crot* genotypes x five mice liver samples x two metabolite extract replicates for each supernatant and pellet fraction (Supplementary Figure S1). We also generated two types of quality controls (QC). The first was the QC-group, a pool for each supernatant vs pellet fraction (liver) and upper vs lower fraction (plasma) per mouse genotype. Each QC-group sample was analyzed using data-dependent acquisition (ddMS2). The combined data were used to generate a small molecule spectral library (Section 2.7). The second was the QC-all sample that represented all genotypes and fractions. This QC was used to monitor MS1 peak integrity throughout the acquisition period. It was injected every five samples generating seven to eight QC samples per analysis. We also collected a mock/blank sample at the beginning of each acquisition batch to account for background molecules and contaminants that may have been carried over across acquisitions (Section 2.7). For each set of metabolite fractions and on-line separation method, the samples were randomized for injection.

*Mass spectrometer settings*—The metabolites were analyzed with the Orbitrap Exploris 480 mass spectrometer fronted with a FLEX ion source coupled to Vanquish Horizon UHPLC system (Thermo Fisher Scientific) using either a reverse phase column (RP, Accucore™ Vanquish™ C18+ UHPLC Column, 1.5 μm, 100 × 2.1 mm; Thermo Fisher Scientific) or a hydrophilic interaction chromatography column (Accucore™ 150 Amide HILIC LC Column, 2.6 μm, 100 × 2.1 mm; Thermo Fisher Scientific) in either positive or negative ion mode for each analytical column. *The RP analytical* gradient flow rate was 0.3 mL/min from 0% to 25% solvent B (methanol/0.1% formic acid) for 4 minutes, 25%–98% solvent B for 4 minutes, and 98% solvent B for 4 minutes. Solvent A was 0.1% formic acid. *The HILIC gradient* flow rate was 0.3 mL/min from 10% to 50% solvent A (2 mM ammonium acetate) for 8 minutes, 50%–90% solvent A for 1 minute, and 10% solvent A for 3 minutes. Solvent B was acetonitrile. For both RP and HILIC runs, the individual samples were analyzed using MS1 scans alone to maximize the number of measurements across the chromatographic peaks since the small molecule library was created using the ddMS2 scans from the QC-group (below). The mass spectrometer was set to 3000 V for positive and 3000 V for negative. For MS1 scans, the instrument was set to 120 K resolution, with a scan range of m/z 70–700, RF lens set to 30%, automatic gain control (AGC) set to standard with the maximum injection time set to auto, and EASY-IC™ calibration enabled. For ddMS2 acquisitions on the QC-group samples, the top N precursor ions in a 1 s cycle time (within a scan range of m/z 70–700; isolation window of m/z 2) were subjected to higher energy collision dissociation (HCD, collision energy 15, 30% and 45%) using 15 K resolution for the MS/MS scans. Dynamic exclusion was enabled (2.5 s). AcquireX’s “Deep Scan” mode (Thermo Fisher Scientific) analysis was applied to the positive ion mode acquisitions for RP analytical gradients.

### 2.6 Small molecule inference

The ddMS2 spectra were analyzed using Compound Discoverer v3.2 (CD, Thermo Fisher Scientific). The “Untargeted Metabolomics” workflow was enabled, and includes feature retention time alignment, unknown compound detection, and compound grouping across all samples. Specifically, the workflow predicts elemental composition for all candidate compounds, fills gaps across all samples and excludes features identified from the mock/blank. Candidate compounds were determined by a similarity search using the MS/MS data against mzCloud, Metabolika, Predicted Compositions, and ChemSpider via CD). Additionally, the workflow applies its mzLogic algorithm to rank order ChemSpider results. This study prioritized metabolites identified as “full match” in mzCloud. For quantification, the QC-all MS1 injections were used for batch normalization (e.g., Supplementary Figures S2, S3) and the Differential Analysis and Normalized Areas (median-normalization) nodes were also applied. The blanks exhibited background signal two-orders magnitude less than any of the QC or sample files (Supplementary Figure S2). We also screened the blanks for potential background contamination across runs. Example molecules demonstrate that they are either absent in blanks, or more than two to three orders of magnitude lower in signal compared to the QC and individual samples (e.g., Supplementary Figure S4).

### 2.7 Mass spectrometry for targeted metabolite profiling

Plasma and liver samples were analyzed by targeted mass spectrometry for L-carnitine, octanoylcarnitine, palmitoylcarnitine, L-carnitine-*d3* (Cayman), octanoylcarnitine-*d3* (Cayman), and palmitoylcarnitine-*d3* (Cayman), using a Orbitrap Exploris 480 coupled to a Vanquish UHPLC systems (Thermo Fisher Scientific). Compounds were separated by an Accucore Vanquish C18+ UHPLC column (100 mm × 2.1 mm, 1.5 μm, Thermo Scientific) heated at 55°C. The gradient flow rate was 0.300 mL/min from 0% to 5% solvent B (0.1% formic acid in methanol) for 1 minute, 5%–98% solvent B for 2 minutes, and 98% solvent B for 3 min. Solvent A was 0.1% formic acid in water. The mass spectrometer was operated in positive ion mode setting to 3000 V. Precursor m/z values were 162.11 for L-carnitine (retention time = 0.6 min), 288.21 for octanoylcarnitine (3.4 min), 400.34 for palmitoylcarnitine (4.0 min), 165.13 for L-carnitine-*d3* (0.6 min), 291.24 for octanoylcarnitine-*d3* (3.4 min), and 403.36 for palmitoylcarnitine-*d3* (4.0 min). The MS/MS spectra (HCD; collision energy, 30%, 40% and 50%) were scanned at 15K resolution (Orbitrap).

Targeted MS/MS data were quantified with Skyline (version 21.1.0.146) (Adams et al., 2020). Peak area ratio (L-carnitine/L-carnitine-*d3*, octanoylcarnitine/octanoylcarnitine-*d3*, and palmitoylcarnitine/palmitoylcarnitine-*d3*) were calculated using the following ions: L-carnitine, m/z 103.04; octanoylcarnitine, m/z 85.03; palmitoylcarnitine, m/z 85.03; L-carnitine-*d3*, m/z 103.039; octanoylcarnitine-*d3*, m/z 85.03; and palmitoylcarnitine-*d3*, m/z 85.03. Standard curves for carnitine-*d3*, octanoylcarnitine-*d3* and palmitoylcarnitine-*d3* were constructed by plotting peak area (y) vs. nominal concentration of standards (x).

### 2.8 Statistical analysis

Kruskal–Wallis test followed by Benjamini Hochberg test was performed using Prism 8 (GraphPad, La Jolla, CA). For box plots, the median-normalized area values (*n* = 5 per genotype) that were calculated by Compound Discoverer v3.2 were used, and graphs were made using Prism eight and Microsoft Excel. Adjusted *p* values were calculated by Compound Discoverer v3.2. Boxplots are metabolites detected in a representative extraction fraction when applicable.

## 3 Results

### 3.1 Study workflow

**FIGURE 1:**
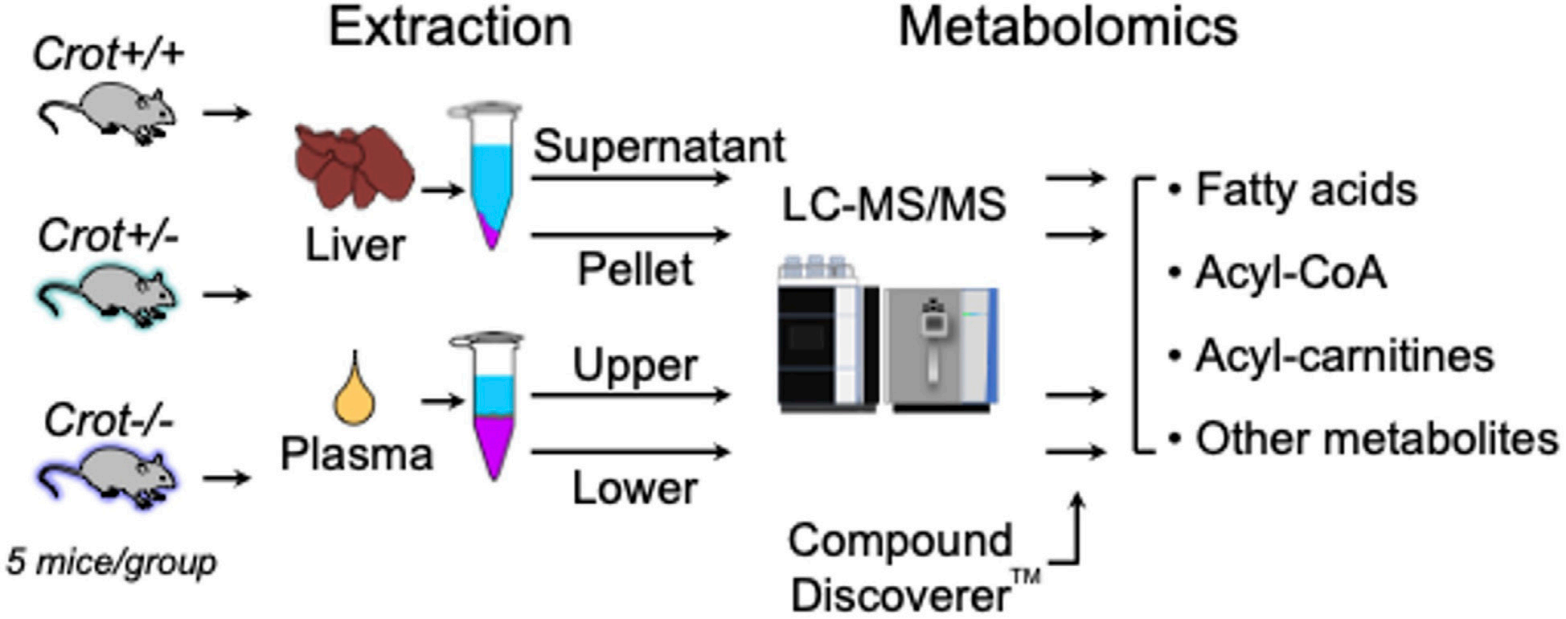
Study flow chart. LC-MS/MS-based metabolomics of liver and plasma samples derived from *Crot+/+*, *Crot+/−*, and *Crot−/−* mice. Metabolites were analyzed using a benchtop quadrupole mass spectrometer and identified using Compound Discoverer™.

To evaluate the impact of CROT deficiency in mouse liver and plasma, we employed LC-MS/MS-based metabolite profiling of *Crot−/−*, *Crot* +/− and *Crot+/+* mice (Figure 1). The metabolites were extracted using a modified dichloromethane-methanol strategy (Ferrarini et al., 2019), that yields a range of hydrophilic (supernatant or upper fraction) and hydrophobic (pellet or lower fraction) for higher and lower polarity compounds, respectively (Figure 1; Supplementary Figure S1).

### 3.2 Global mouse liver and plasma metabolite profiles

**FIGURE 2:**
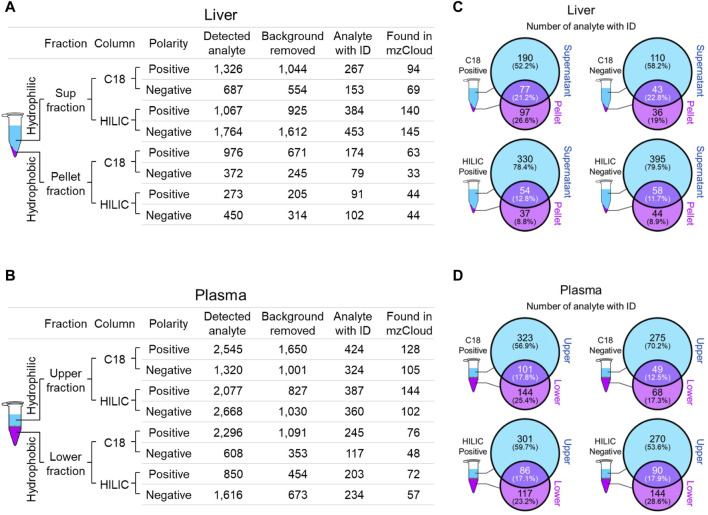
Overview of identified liver and plasma metabolites. **(A and B)**, Number of detected and annotated analytes for liver **(A)** and plasma **(B)** samples. Metabolite fractionation columns are indicated (C18, hydrophobic; HILIC, hydrophilic), as is the instrument polarity. Detected analytes were filtered (Background removed) for those identified in the mock control. Candidate analyte identifications (Analyte with ID) were inferred from one of four queried annotation sources, but only those with a full match in mzCloud (Found in mzCloud) were further prioritized. **(C and D)**, The extent of overlap between each fraction was evaluated using analytes with candidate IDs from liver **(C)** and plasma **(D)**.

We employed two chromatographic fractionation methods, reverse-phase and hydrophilic interaction liquid chromatography (HILIC), to optimize the separation of increasingly hydrophobic and hydrophilic metabolites, respectively. Each fractionated sample was then analyzed in either positive or negative polarity mode to optimize identification of the metabolites in their preferred ion state. Figures 2A,B summarizes the filtering of mass spectral data from the number of detected analytes to those with a putative identification from at least one of four annotation sources, and then to those with a full match in the mzCloud spectral library. The number of mzCloud matches ranged from 33 to 145 for the combined (three *Crot* genotypes) liver profiles (Figure 2A), and from 48 to 144 for the combined plasma profiles (Figure 2B). For both liver and plasma analyses, the corresponding hydrophilic fractions yielded more metabolites than their respective hydrophobic fractions. The hydrophobic fractions nonetheless provided unique metabolites, ranging from 8.8% to 29.6% and 17.3% and 28.6% for total liver and plasma metabolites, respectively (Figures 2C,D).

### 3.3 CROT deficiency increased omega-3 fatty acids in the liver

**FIGURE 3:**
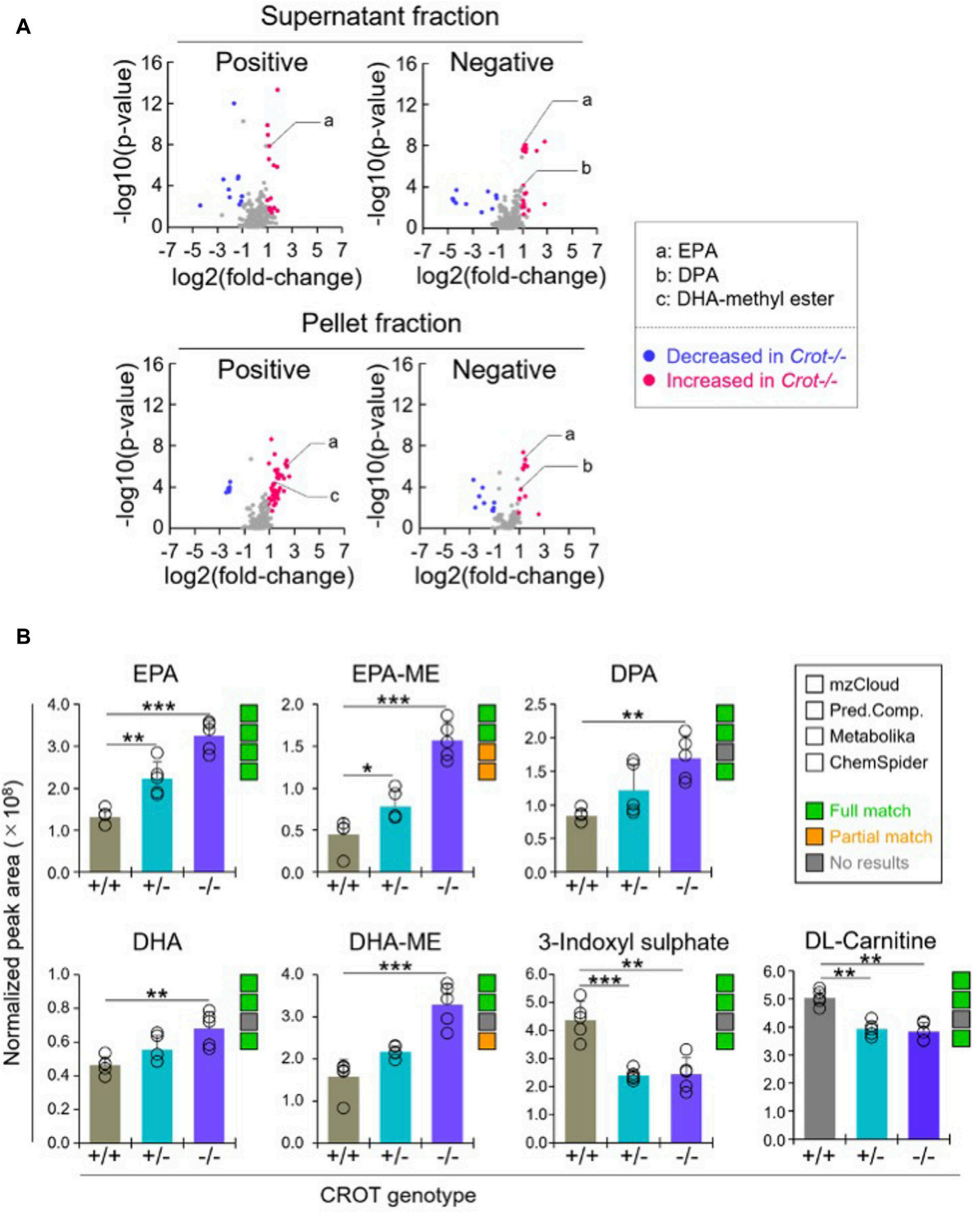
Differential analysis of detected metabolites in liver. **(A)**, Representative volcano plots for the comparison between *Crot−/−* versus *Crot+/+* mice liver samples. Log2 fold-change>1 or < −1, *p* < 0.05) were labeled with red or blue, respectively. **(B)**, Bar graphs demonstrating the peak areas of featured metabolite (EPA: eicosapentaenoic acid, DPA: docosapentaenoic acid, DHA: docosahexaenoic acid, DHA-ME: DHA-methyl ester, three-indoxyl sulphate, and DL-carnitine) that were median-normalized in Compound Discoverer. **: *p* < 0.01, ***: *p* < 0.001, Kruskal–Wallis test followed by Benjamini Hochberg test (n = 5). Status of metabolite identification in each database (mzCloud, Predicted Compositions, Metabolika, and ChemSpider) was shown with green (full match), orange (partial match), and grey (no results).

To identify liver metabolites that were altered by CROT deficiency, we performed differential abundance analysis between *Crot−/−* and *Crot+/+* mice using all candidate metabolites (“Analytes with ID,” Figure 2A). We filtered for metabolites that increased or decreased (*p* < 0.05; q < 0.30) for each analytical condition—fraction, column, and polarity (Figure 3A; Supplementary Figure S5A), and then focused subsequent analysis on metabolites that with full matches in mzCloud (Supplementary Table S1). When considering all analytical conditions, 21 and 48 metabolites increased or decreased, respectively, with CROT deficiency (Table 1; Supplementary Table S1). Some metabolites were measured in more than one analytical condition providing quantification replicates. For example, the omega-3 fatty acid eicosapentaenoic acid (EPA) (Supplementary Figure S6), was identified in the supernatant and pellet fractions (Supplementary Table S1). EPA and other omega-3 fatty acids and derivates such as, EPA methyl ester (EPA-ME), docosahexaenoic acid (DHA), DHA methyl ester (DHA-ME), and docosapentaenoic acid (DPA), increased in *Crot−/−* (Figure 3B; Table 1; Supplementary Table S1). We also noted that some of these fatty acids increased in a CROT*-*deficient manner (Figure 3B). Notable metabolites that decreased with CROT deficiency include three-indoxyl sulphate, omega-1 hydroxyhexadecanoic (hydroxypalmitic) acid, omega-6 docosadienoic acid, betaine, and L-carnitine (Figure 3B; Supplementary Table S1).

**TABLE 1:** Featured differentially abundant liver metabolites in *Crot*−/− vs. *Crot*+/+ mice. Metabolites are from the Volcano plots in Figure 3. Four of the top-10 differentially abundant (increased or decreased, by *p*-value) metabolites are featured. Positive values (increased in *Crot*−/− mice), negative values (decreased in *Crot*−/− mice).

Metabolite	*p*-value	Fold-change [log2] Crot−/− vs Crot +/+	Column	Ion mode	
Pregabalin	5.47E-14	1.85	C18	Positive	Supernatant
Cipamfylline	1.15E-12	−1.61	C18		
Dihydrothymine	6.17E-11	−0.91	C18		
Eicosapentanoic acid	1.65E-08	1.19	C18		
cis-5, 8, 11, 14, 17-Eicosapentaenoic acid	1.80E-08	1.18	C18	Negative	
Docosapentaenoic acid	8.61E-05	1.07	C18		
Erucic acid	9.19E-04	−0.4	C18		
Stearidonic acid	1.04E-03	0.79	C18		
Eicosapentaenoic acid methyl ester	1.07E-06	2.45	C18	Positive	Pellet
Docosatetraenoylethanolamide	1.30E-05	1.66	C18		
Virodhamine	1.64E-05	1.76	C18		
Docosahexaenoic acid methyl ester	5.65E-05	1.38	C18		
cis-5, 8, 11, 14, 17-Eicosapentaenoic acid	2.38E-07	1.49	C18	Negative	
Erucic acid	1.49E-04	−0.64	C18		
Docosapentaenoic acid	2.05E-04	1.14	C18		
13Z,16Z-Docosadienoic Acid	3.52E-03	−1	C18		
gamma-Valerolactone	2.65E-14	2.2	HILIC	Positive	Supernatant
(2S)-2-Amino-8-hydroxyoctanoic acid	2.44E-13	1.26	HILIC		
Pentahomoserine	1.65E-11	−1.49	HILIC		
Salinosporamide D	1.99E-09	−2.78	HILIC		
Eicosapentaenoic acid	1.28E-08	1.17	HILIC	Negative	
p-Cresylsulfate	1.37E-07	−2.1	HILIC		
Taurine	2.31E-07	1.7	HILIC		
Ethylmalonic acid	1.95E-05	−0.93	HILIC		
O-adipoylcarnitine	7.18E-06	−1.87	HILIC	Positive	Pellet
N-Stearoyltyrosine	1.24E-03	−1.34	HILIC		
Bethanidine	1.70E-03	−1.4	HILIC		
Panthenol	2.47E-03	1.88	HILIC		
Eicosapentaenoic acid	3.03E-10	1.53	HILIC	Negative	
Docosapentaenoic acid	6.00E-05	0.96	HILIC		
3, 6-Anhydro-1-O-palmitoylhexitol	6.02E-04	0.94	HILIC		
Docosahexaenoic acid	4.97E-03	0.64	HILIC		

### 3.4 CROT deficiency increased dicarboxylic acids in plasma

**FIGURE 4:**
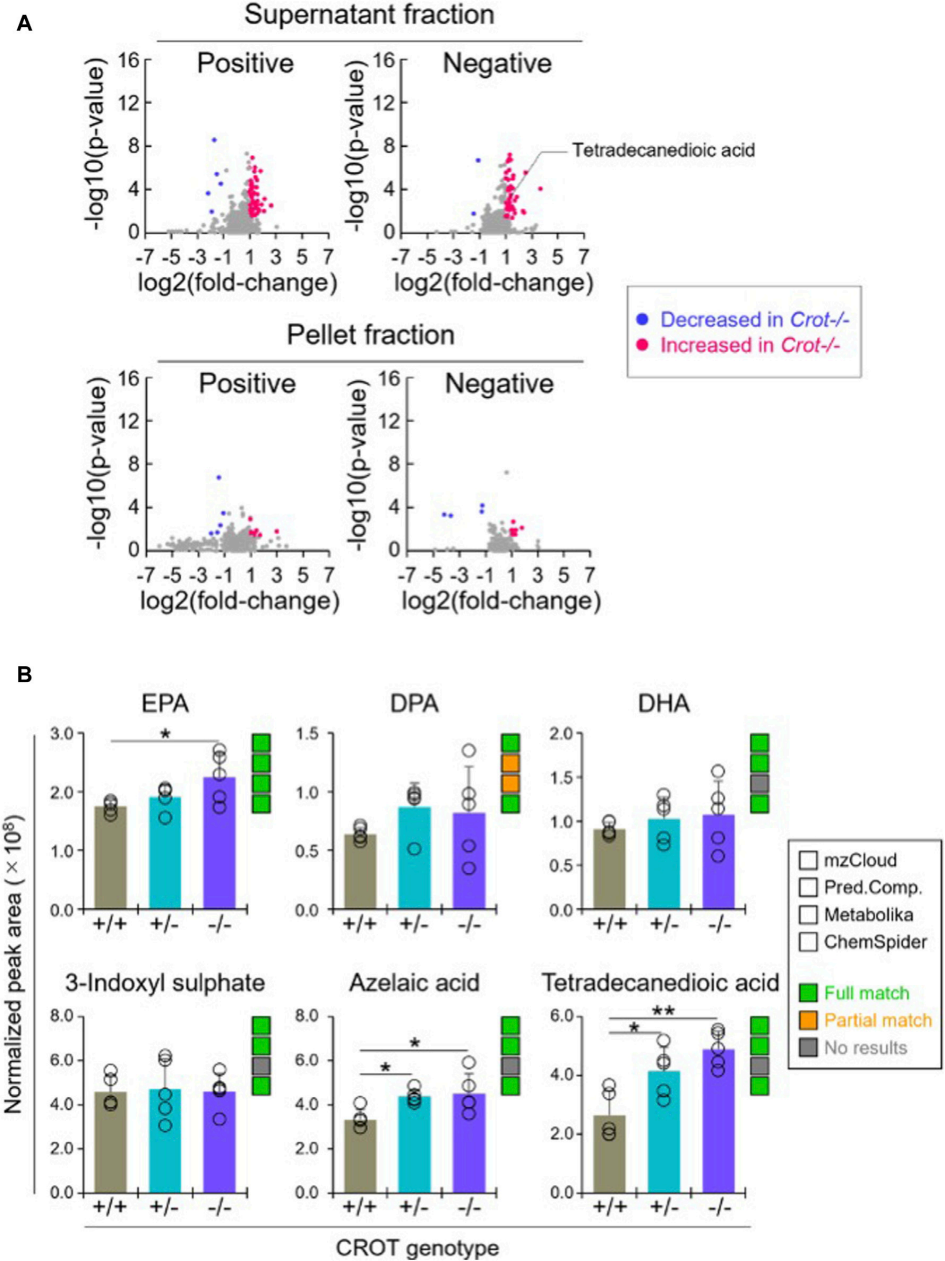
Differential analysis of detected metabolites in plasma. **(A)**, Representative volcano plots for the comparison between *Crot−/−* versus *Crot+/+* mice plasma samples. Log2 fold-change>1 or < −1, *p* < 0.05) were labeled with red or blue, respectively. **(B)**, Bar graphs demonstrating the peak areas of featured metabolite (EPA: eicosapentaenoic acid, DPA: docosapentaenoic acid, DHA: docosahexaenoic acid, three-indoxyl sulphate, azelaic acid, and tetradecanedioic acid) that were median-normalized in Compound Discoverer. *: *p* < 0.05, **: *p* < 0.01, Kruskal–Wallis test followed by Benjamini Hochberg test (n = 5). Status of metabolite identification in each database (mzCloud, Predicted Compositions, Metabolika, and ChemSpider) was shown with green (full match), orange (partial match), and grey (no results).

When applying the same differential abundance analysis strategy as liver (*Crot−/−* vs *Crot+/+*; *p* < 0.05; *q* < 0.30), fewer plasma metabolites with full matches in mzCloud remained; 11 increased and 3 decreased with CROT deficiency (Table 2; Supplementary Table S2). In addition, most of the metabolites were identified in the supernatant fraction of the extraction protocol (Figure 4A; Supplementary Figure S5B). Although EPA, DPA, and DHA were detected, relative to the liver, these metabolites were stable in CROT-deficient plasma (Figure 4B). Interestingly, two of the increased metabolites are members of the dicarboxylic acids, tetradecanedioic acid and azelaic acid (Figure 4B; Supplementary Figure S7). Taurine, cytosine and triethanolamine were the three metabolites that decreased in plasma of *Crot−/−* mice (Supplementary Table S2).

**TABLE 2:** Featured differentially abundant plasma metabolites in *Crot*−/− vs. *Crot*+/+ mice. Metabolites are from the Volcano plots in Figure 4. Four of the top-10 differentially abundant (increased or decreased, by *p*-value) metabolites are featured. Positive values (increased in *Crot*−/− mice), negative values (decreased in *Crot*−/− mice).

Metabolite	*p*-value	Fold-change [log2] Crot-/- vs Crot +/+	Column	Ion mode	
Z-Leu-OH	3.19E-09	−1.67	C18	Positive	Supernatant
1-(beta-D-ribofuranosyl)thymine	2.22E-06	1.87	C18		
3, 5-Tetradecadiencarnitine	3.34E-05	−1.17	C18		
Tiglylcarnitine	5.51E-05	1.23	C18		
Xanthosine	4.69E-02	1	C18	Negative	
Traumatic Acid	8.86E-05	1.56	C18		
Tiglylcarnitine	2.94E-02	0.82	C18		
Tetradecanedioic acid	1.65E-03	1.31	C18		
Triethanolamine	4.80E-02	−0.97	C18	Positive	Pellet
triamterene	4.87E-03	−1.28	C18		
testosterone ketolaurate	3.86E-02	1.24	C18		
Pipecolic acid	3.43E-02	0.14	C18		
Trifluoroacetic acid	4.85E-04	−0.4	C18	Negative	
Tetradecanedioic acid	1.32E-02	−4.15	C18		
Taurine	8.55E-03	1.34	C18		
Propapyriogenin A2	3.27E-02	−0.44	C18		
Zalcitabine	1.49E-03	1.09	HILIC	Positive	Supernatant
Taurine	3.91E-03	−0.21	HILIC		
salinosporamide D	5.64E-10	−0.25	HILIC		
promolate	4.21E-02	−1.97	HILIC		
trans-geranic acid	2.28E-02	0.45	HILIC	Negative	
Tetradecanedioic acid	1.16E-03	0.81	HILIC		
Taurine	1.64E-03	0.66	HILIC		
oleandolide	6.72E-07	−0.27	HILIC		
Triethanolamine	1.01E-02	0.98	HILIC	Positive	Pellet
Taurine	3.50E-03	−0.82	HILIC		
salinosporamide D	6.65E-09	−0.39	HILIC		
N-Stearoyltyrosine	1.26E-04	−1.73	HILIC		
Trifluoroacetic acid	2.25E-04	0.7	HILIC	Negative	
prohydrojasmon	9.86E-04	−3.7	HILIC		
Palmitic acid	3.05E-02	1.06	HILIC		
oleandolide	6.42E-06	−0.1	HILIC		

### 3.5 Liver vs plasma acylcarnitine and carnitine levels varied in response to CROT deficiency

**FIGURE 5:**
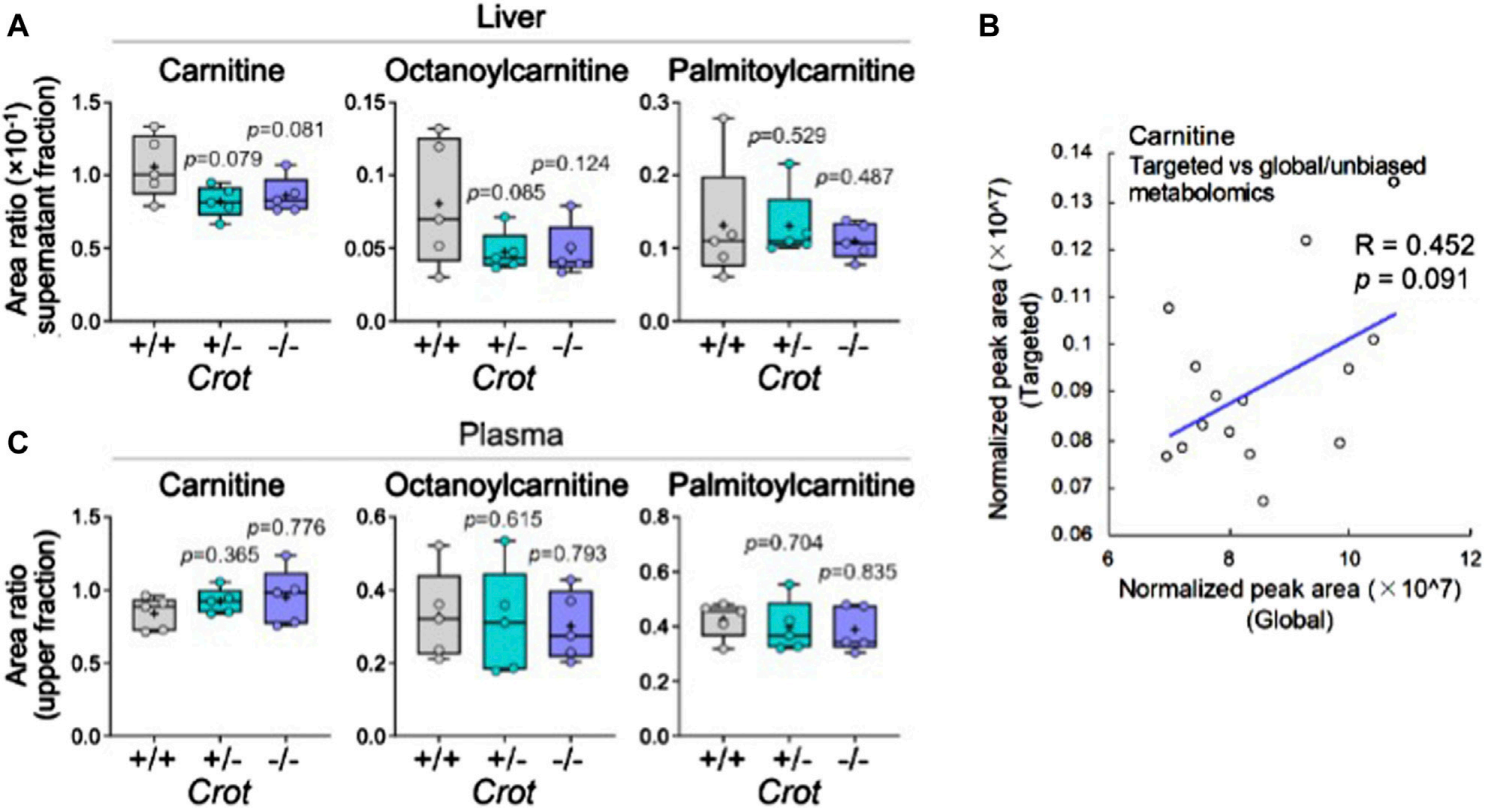
L-carnitine and acyl-carnitine targeted MS2 mouse liver data. **(A)** Box plots summarizing targeted mass spectrometry results for L-carnitine and acyl-carnitines measured in liver. Kruskal–Wallis test followed by Benjamini Hochberg test was performed (n = 5). **(B)** Correlation between global and targeted metabolomics of liver carnitine. Pearson’s correlation coefficient (R) and *p*-value are shown. **(C)** Box plots summarizing targeted mass spectrometry results for L-carnitine and acyl-carnitines measured in plasma. Kruskal–Wallis test followed by Benjamini Hochberg test was performed (n = 5).

CROT catalyzes the carnitinylation of octanoyl-CoA and palmitoyl-CoA to octanoylcarnitine and palmitoylcarnitine. Carnitine (DL-carnitine) was detected in the global metabolomics analysis—whereas its levels did not change in plasma it did decrease in the liver of both *Crot* +/− and *Crot−/−* mice compared to *Crot+/+* (Supplementary Table S1, *p* < 0.002; q < 0.025). We employed a targeted MS analysis strategy to specifically measure the L-carnitine isomer in addition to octanoylcarnitine and palmitoylcarnitine since they were not detected in the global metabolomic analysis (Supplementary Figure S8A; Supplementary Table S3). In liver, L-carnitine showed a tendency to decrease (Figure 5A), consistent with the global metabolomics (Figure 5B). On the other hand, plasma L-carnitine did not decrease with CROT-deficiency (Figure 5C). Octanoylcarnitine decreased in liver only (Figure 5C; *p* = 0.094 and *p* = 0.117 for *Crot* +/− and *Crot*−/−, respectively); whereas palmitoylcarnitine levels remained constant across the 3 mouse genotypes for liver and plasma (Figures 5A,C; Supplementary Figure S8A).

## 4 Discussion

CROT is traditionally studied in the context of peroxisome function and proliferation, and general lipid metabolism (Kunau et al., 1995), but we recently discovered its role in mediating cardiovascular calcification (Okui et al., 2021). How lipid dysfunction specifically promotes calcification potential remains unknown, thus the discovery that CROT inhibition reduces calcification potential in a mouse model for atherosclerosis (Okui et al., 2021) provided the rationale to assess more carefully CROT function in normolipidemic mice in the present study.

The liver is a central organ for gluconeogenesis, detoxification, and lipid metabolism. Lipids in turn are secreted and enter circulation to reach target organs. We therefore prioritized profiling the metabolites of liver and plasma compartments of CROT-deficient mice. Unbiased metabolite profiling demonstrated an increase of omega-3 fatty acids, EPA [20:5 (n-3)], EPA-ME, DHA [22:5 (n-3)], DHA-ME, and DPA [22:6 (n-3) in liver. Our previous study, that instead employed a targeted lipid panel, demonstrated an increase in EPA and a tendency of increase for DPA in CROT siRNA-treated human coronary artery smooth muscle cells (Okui et al., 2021). EPA and DPA are synthesized from α-linolenic acid [ALA (18:3 (n-3)] via the desaturase/elongase pathway in the endoplasmic reticulum (Burdge, 2006); but the final step in DHA synthesis is uniquely via retroconversion (β-oxidation) of tetracosahexaenoic acid [24:6 (n-3)] in the peroxisome (Moore et al., 1995; Masters, 1996; Ferdinandusse et al., 2001; Burdge, 2006). CROT inhibition may have therefore favored a shift of fatty acids away from catabolism by carnitine-dependent β-oxidation by the mitochondria and towards anabolism via release of free fatty acids into the cytosol. Despite the favorable shift towards omega-3 polyunsaturated fatty acids with CROT-deficiency, this increase alone may be inconsequential to potential anti-inflammatory-dependent mitigation of vascular calcification. Although EPA and DHA can be synthesized from ALA, they are nonetheless considered essential fatty acids, acquired through the consumption of, for instance, fish and nuts. Previous fish oil or omega-3 polyunsaturated fatty acid feeding studies in human and animals, demonstrated significant decreases in inflammation-inducing plasma triglycerides (Sanders and Roshanai, 1983; Herold and Kinsella, 1986); but such a decrease was dependent on consuming of at least 5 g or 20 mL of PUFA daily in humans (Sanders and Roshanai, 1983; Herold and Kinsella, 1986). Moreover, these early feeding studies were not designed to determine whether omega-3 fatty acids reduced cardiovascular risk.

Several studies have proposed that dietary omega-3 fatty acids protect against cardiovascular disease by lowering risk factors such as blood pressure and heart rate, serum triglycerides, thrombotic tendency, inflammation, and arrhythmias, but also by improving endothelial function, insulin sensitivity, paraoxonase activity, and plaque stability (Ferdinandusse et al., 2001; Masters, 1996; von Schacky and Harris, 2007; Hooper et al., 2006). However, despite a century of reports investigating the potential health benefits and medicinal properties of omega-3 polyunsaturated fatty acids (Guha et al., 1930; Herold and Kinsella, 1986), recent large cohort cardiovascular clinical trials have yielded inconsistent findings (Sherratt et al., 2023). An ethyl ester formulation of EPA known as icosapent ethyl (IPE), but not traditional carboxylic acid formulations of mixed omega-3 fatty acids, reduced cardiovascular events in patients with controlled low-density lipoprotein-cholesterol (LDL-C) levels (Mason and Eckel, 2021). The proposed reasons for these discordant findings are numerous and debatable, including DHA negating the beneficial effects of EPA (Sharma et al., 2020; Mason and Eckel, 2021). Interestingly, in the IPE trials, the reduction in cardiovascular events was independent of triglyceride-lowering; suggesting pleiotropic actions independent of plasma triglyceride metabolism (Sharma et al., 2020; Mason and Eckel, 2021).

In the present study, CROT inhibition also resulted in a decrease in omega-1 hydroxyhexadecanoic acid (hydroxypalmitic) and omega-6 docosadienoic acid [22:2 (n-6)] suggesting a potential feedback mechanism to decrease other fatty acid levels when omega-3 acids are increased. Whether CROT activity feeds into a balance of fatty acid classes (omega-3 vs. omega-1, omega-6), however, is not known and would require a targeted analysis to evaluate a broader range of fatty acids. Another study investigated the impact of CROT inhibition on HepG2 cell lipids using a targeted analysis, and found that EPA, DPA, and DHA were not changed (Le Borgne et al., 2011). Instead, very long omega-6, saturated and monounsaturated fatty acids increased (Le Borgne et al., 2011), pointing to potential differences in fatty acid metabolism between cell types or between primary and immortalized cells.

L-carnitine also decreased in response to CROT-deficiency. L-carnitine is an absolute requirement for mitochondrial beta-oxidation system; thus, its decrease may mitigate oxidative phosphorylation (ATP production), a potential contributor to smooth muscle cell calcification potential (Okui et al., 2021). These results point towards future investigations into whether CROT-mediated inhibition of calcification potential correlates with a decrease L-carnitine in vascular tissues.

The observation that two dicarboxylic acids, long-chained tetradecanedioic acid (14 carbons) and medium-chained azelaic acid (9 carbons), increased in plasma with CROT-deficiency is also interesting. Hepatocytes from mice with liver selective elimination of peroxisomes (liver specific Pex5 deficient mice) displayed severely impaired oxidation of tetradecanedioic acid (Dirkx et al., 2007). Taken together, our present results indicate that CROT may contribute to oxidation of tetradecanedioic acid, directly or indirectly. More is known about azelaic acid since it is exhibits anti-inflammatory and anti-bacterial properties and is an FDA-approved drug for the treatment of acne (Gupta and Gover, 2007). Medium-chained dicarboxylic acids such as azelaic acid are formed from the omega-oxidation of monocarboxylic acids, or the beta-oxidation of longer chained dicarboxylic acids and are known to circulate and be excreted in urine (Grego and Mingrone, 1995; Mingrone and Castagneto, 2006). However, relative to free fatty acids and their acyl-counterparts, much less is known about whether dicarboxylic acids are regulated via the peroxisome and CROT.

Our unbiased metabolite profiling provided a range of mostly hydrophilic analytes and only a subset of small or amphipathic lipids, which is not unexpected since no single extraction method can cover all metabolite and lipid classes (Brugger, 2014; Atkins et al., 2020). Other studies, including our own, have relied on targeted fatty acid panels to evaluation CROT function (Le Borgne et al., 2011; Okui et al., 2021). However, the discovery that the dicarboxylic acids were elevated in plasma of CROT-deficient mice underscored the advantage of undertaking untargeted metabolomics as well. On the other hand, our unbiased metabolomics did not detect known CROT products octanoylcarnitine and palmitoylcarnitine. We therefore employed a targeted mass spectrometry strategy, enabled by their stable isotope-labeled lipid forms, to increase their endogenous signals.

## 5 Conclusion

Our findings confirm that genetic deletion of CROT in normolipidemic mice leads to changes in the liver metabolome consistent with known CROT functions. Mainly, omega-3 fatty acids increase in abundance consistent with a shift away from carnitine-dependent catabolic fates of very long-chained precursors, and towards anabolic metabolism in the cytosol. The observation that the anti-inflammatory azelaic acid increases in plasma with CROT deficiency provides us with the rationale to investigate further the metabolism of dicarboxylic acids, with the potential that azelaic acid may serve as a biomarker for CROT activity.

## Data Availability

The data presented in this study were deposited to MassIVE, identifier: MSV000094756 and are available at: https://massive.ucsd.edu/ProteoSAFe/dataset.jsp?task=dd04ba19dc004668ac64fd296f335669.
