# Supplementary material for: Carnitine O-octanoyltransferase (CROT) deficiency in mice leads to an increase of omega-3 fatty acids
Source: Front Mol Biosci. 2024 Jul 15;11:1374316. doi: 10.3389/fmolb.2024.1374316 (PMC11284101; doi:10.3389/fmolb.2024.1374316)
Supplement: Supplementary file 2 [file Image1.pdf]

# Supporting information
## Supplemental figures

Carnitine O-octanoyltransferase deficiency in mice leads to an increase of omega-3 fatty acids

Takehito Okui[1,†], Shiori Kuraoka[1,†], Masaya Iwashita[1], Rei Iwagata[1], Taku Kasai[1], Masanori Aikawa[1,2], Sasha A. Singh[1,*], Elena Aikawa[1,2*]

[1]Center for Interdisciplinary Cardiovascular Sciences, Division of Cardiovascular Medicine, Brigham and Women's Hospital, Harvard Medical School, Boston, MA, 02115, USA
[2]Center for Excellence in Vascular Biology, Cardiovascular Division, Brigham and Women's Hospital, Harvard Medical School, Boston, MA, 02115, USA

[†]These authors contributed equally

**Running title:**
Metabolome alterations by CROT deficiency

***Corresponding authors:**

Elena Aikawa, MD, PhD
Brigham and Women's Hospital, Harvard Medical School,
3 Blackfan Street, 17th Floor, Boston, MA 02115,
Phone: 617-730-7729
eaikawa@bwh.harvard.edu

Sasha A. Singh, PhD
Brigham and Women's Hospital, Harvard Medical School,
3 Blackfan Street, 17th Floor, Boston, MA 02115,
Phone: 617-730-7702
sasingh@bwh.harvard.edu

**Keywords:** CROT, metabolomics, mass spectrometry, acyl-carnitine, knockout mouse

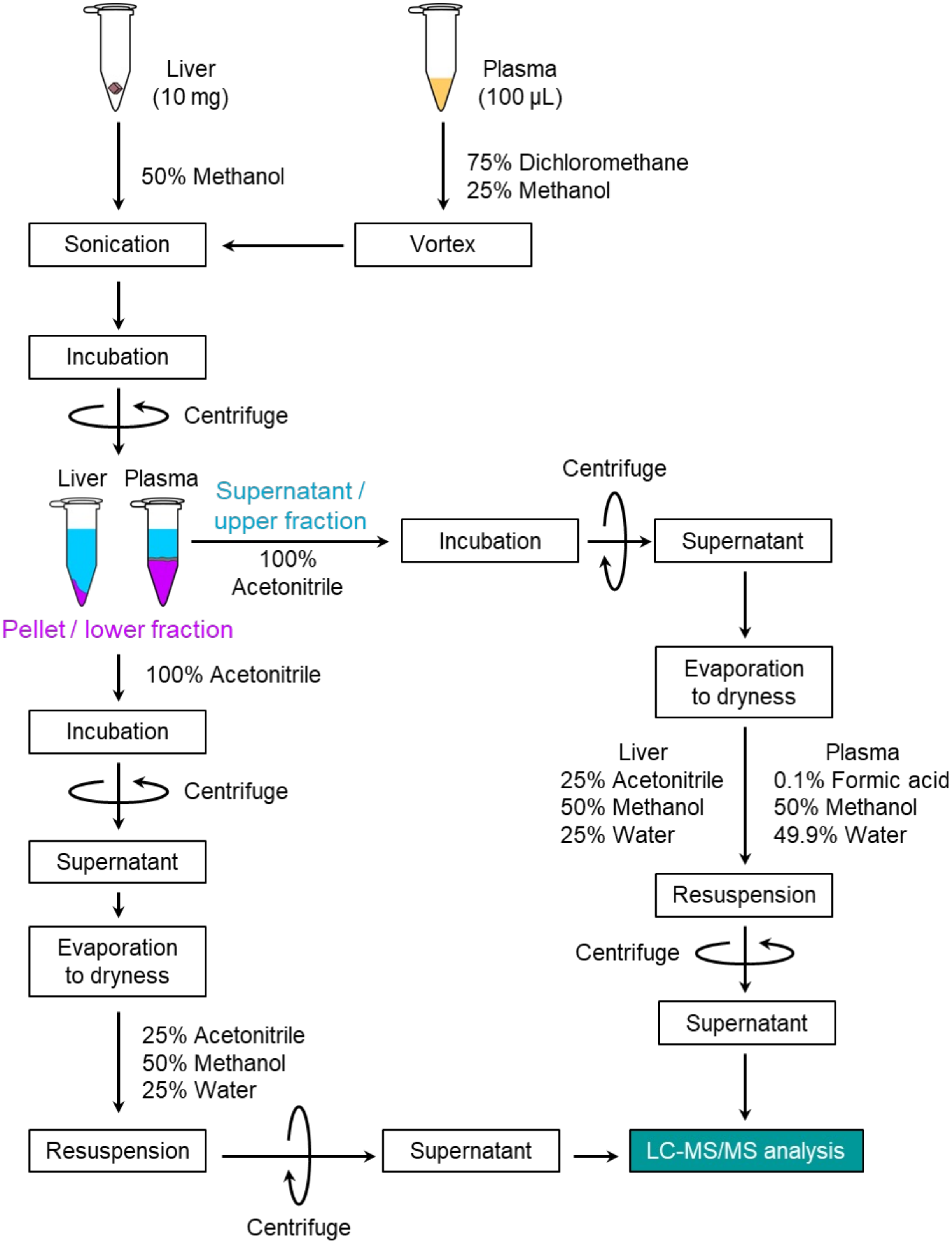


**Supplemental Figure 1.** Flow chart for metabolite extraction protocol.

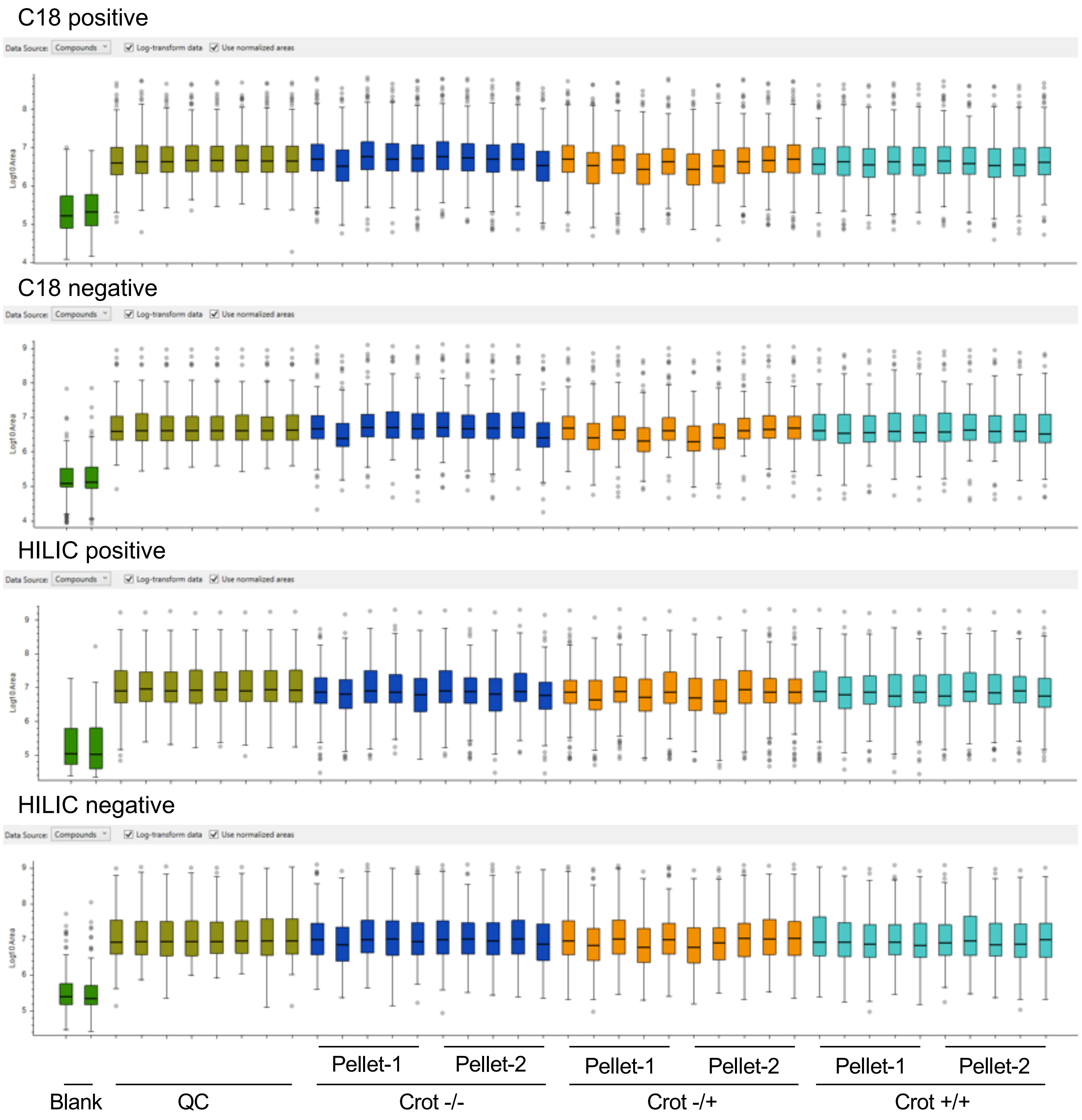


**Supplemental Figure 2**. Example output of quality control (QC-All)-normalized chromatographic peak areas of metabolites derived from mouse liver pellet fractions. To maximize the number of identified metabolites, distinct chromatographic methods (C18, reverse phase vs HILIC) and acquisition methods (positive or negative ion mode) were applied in this study.

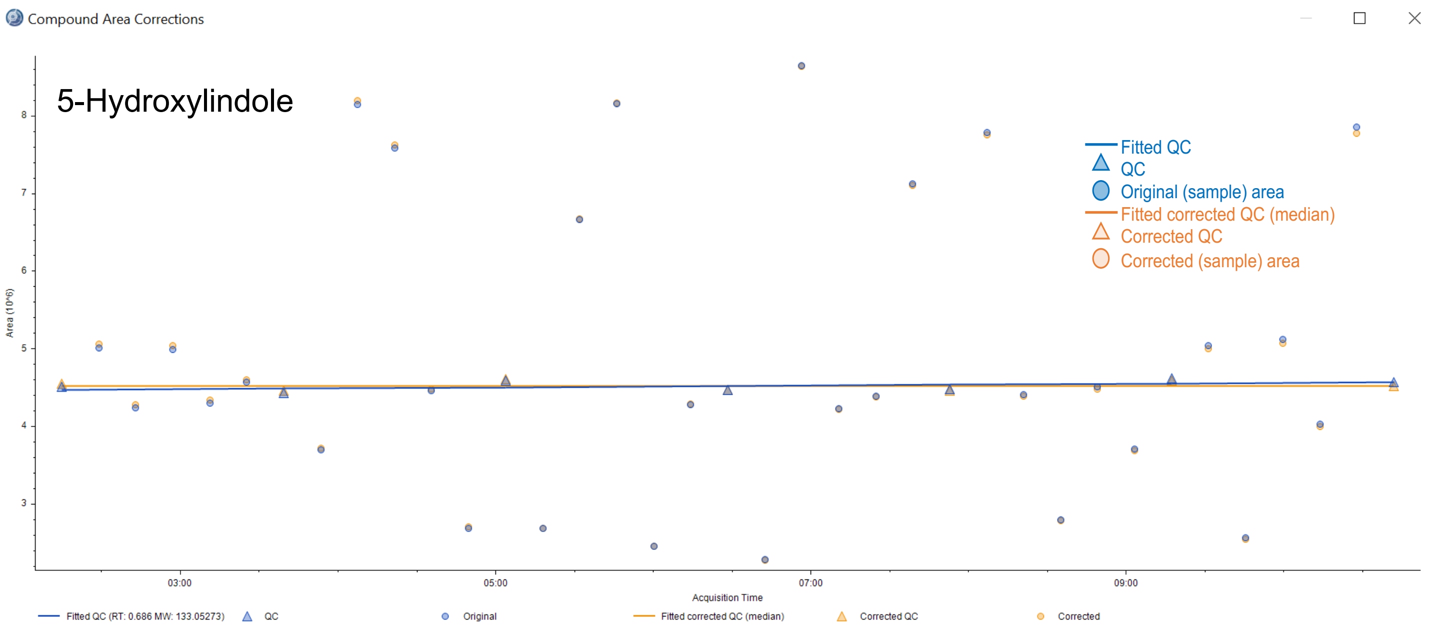


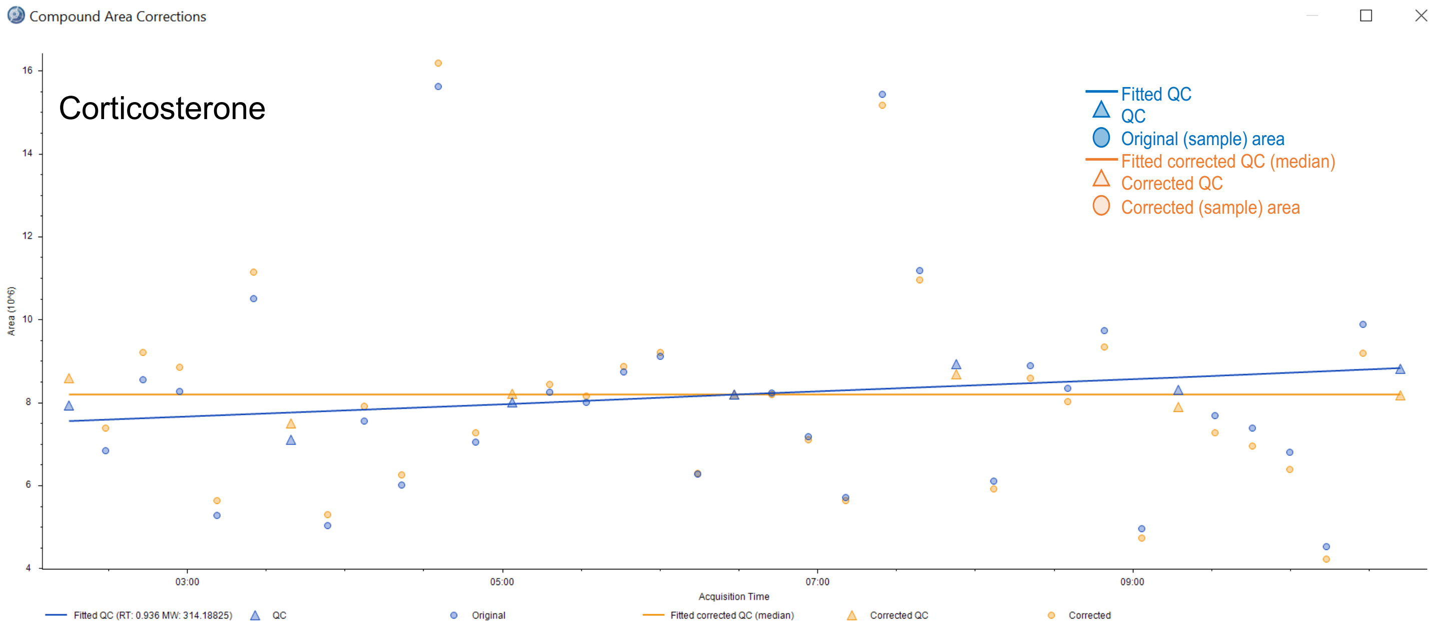


**Supplemental Figure 3**. Example small molecule peak monitoring enabled by the QC-All sample injections (n=7). Each compound was derived from mouse plasma (lower fraction samples, HILIC separation and positive ion mode acquisition). 5-Hydroxylindole was relatively stable throughout acquisition period (about 10 hours); whereas the corticosterone peak increased slightly through the acquisition period.

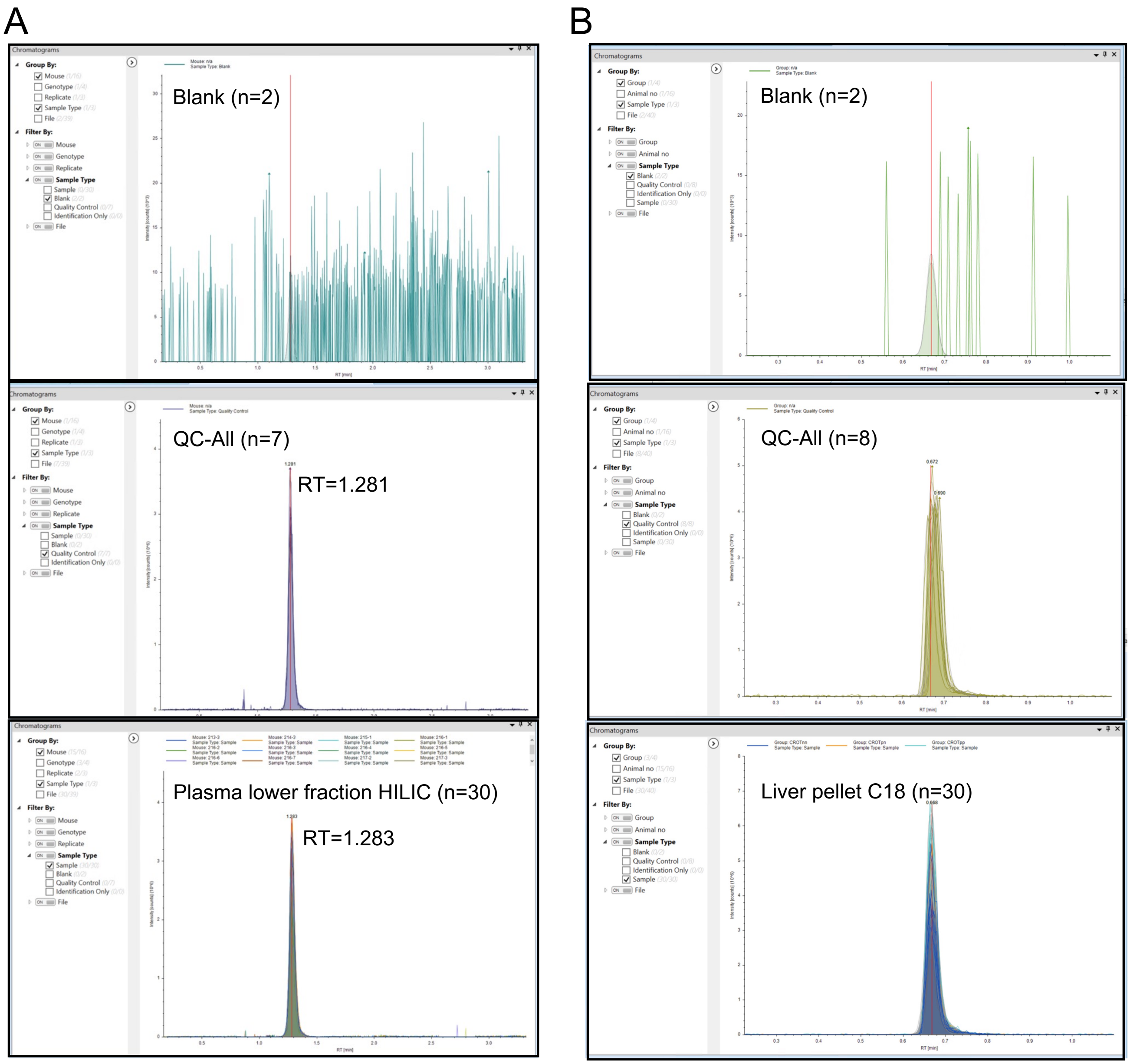


**Supplemental Figure 4**. Extracted MS1 ion chromatograms for A) 4-amino-3-hydroxybuteric acid (C7 H7 N O3) identified in mouse plasma (lower fraction, HILIC, positive ion mode). The peak is not detected in either blank sample but is detected the QC and individual mouse plasma pellet samples. Note that the the number of superimposed chromatograms coincide with the number of samples. B) DL-carnitine (C7 H15 N O3) identified in mouse liver (pellet, C18, positive ion mode)

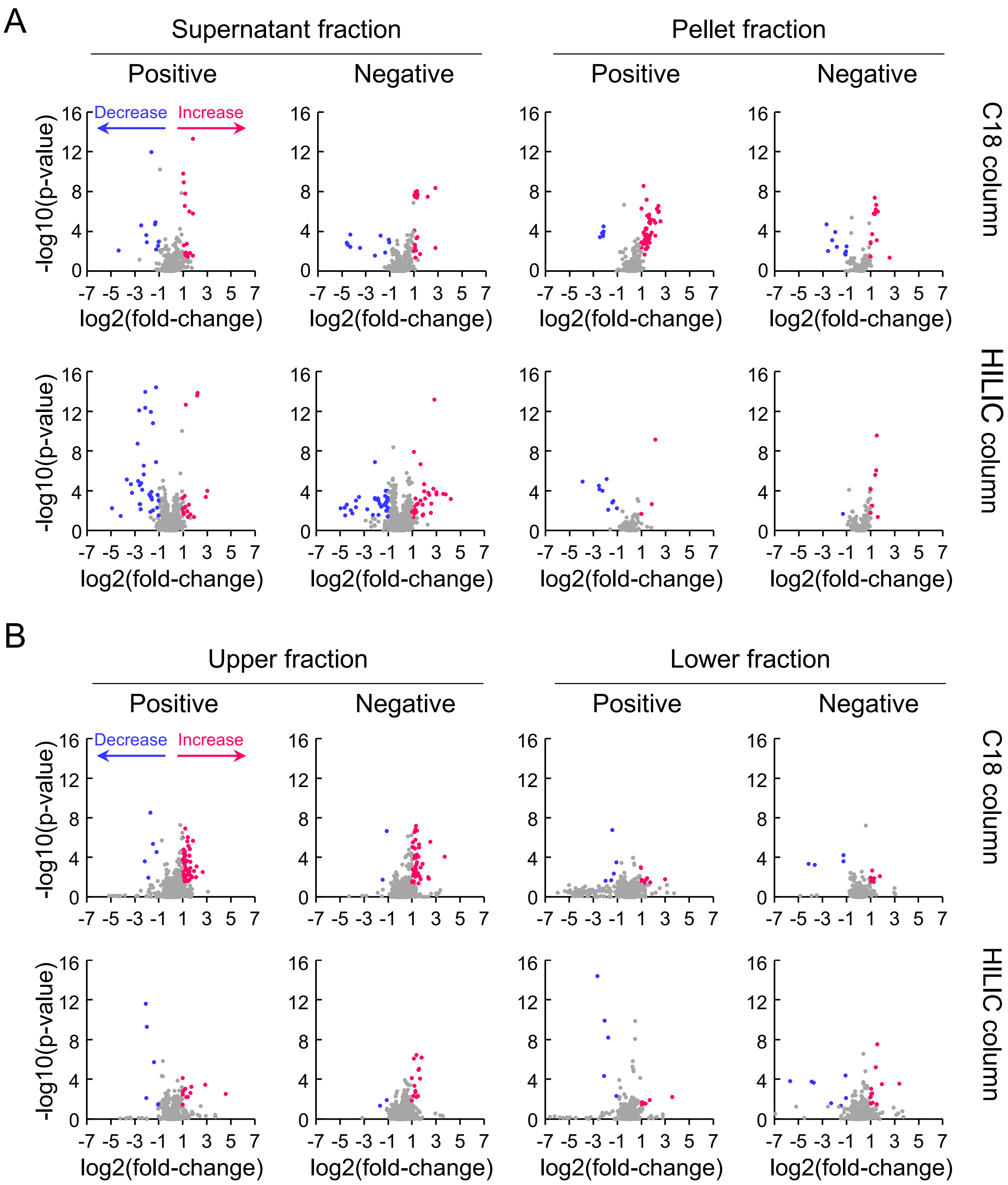


**Supplemental Figure 5**. Volcano plots demonstrating altered liver (A) and plasma (B) metabolites between *Crot+/+* and *Crot-/-* sibling mice (n=5 mice per genotype group). Colored metabolites surpass the log2 fold-change, and p<0.05 thresholds.

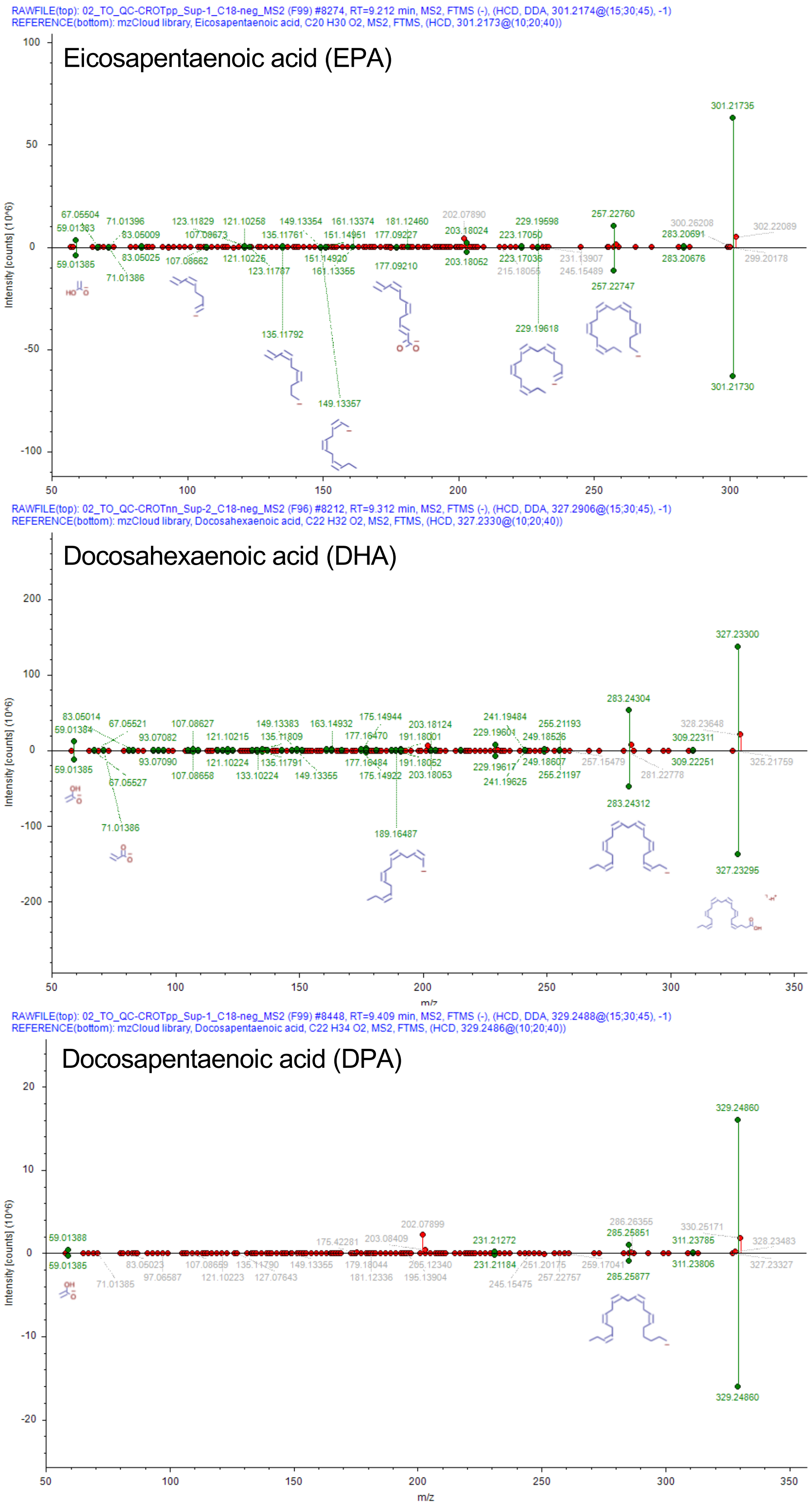


**Supplemental Figure 6**. MS2 spectra for example poly-unsaturated (omega-3) fatty acids identified in mice liver. The mirrored spectrum is the mzCloud reference with annotations. Green, matching spectra; red, non-specific spectra. The headers indicate the quality control condition from which the spectrum was sequenced. CROTpp (*Crot+/+),* CROTnn (*Crot-/-*).

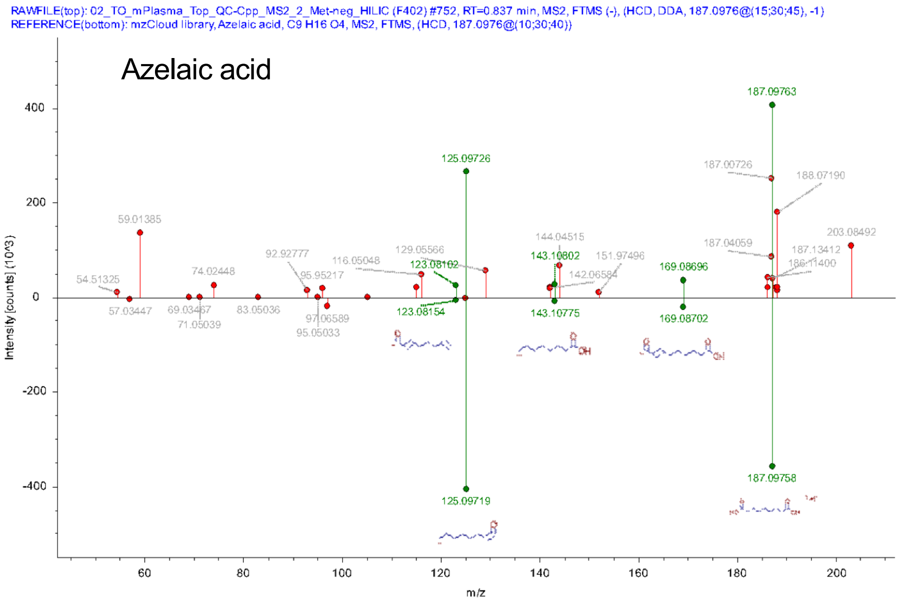


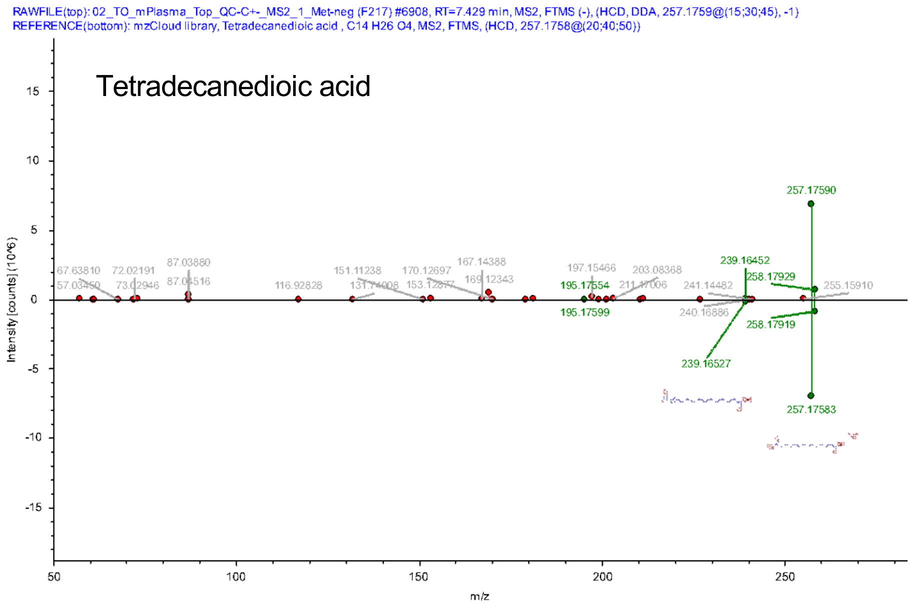


**Supplemental Figure 7**. MS2 spectra for tricarboxylic acids identified in mice plasma. The mirrored spectrum is the mzCloud reference with annotations. Green, matching spectra; red, non-specific spectra. The headers indicate the quality control condition from which the spectrum was sequenced. CROTpp (*Crot+/+*), CROTnn (*Crot-/-*).

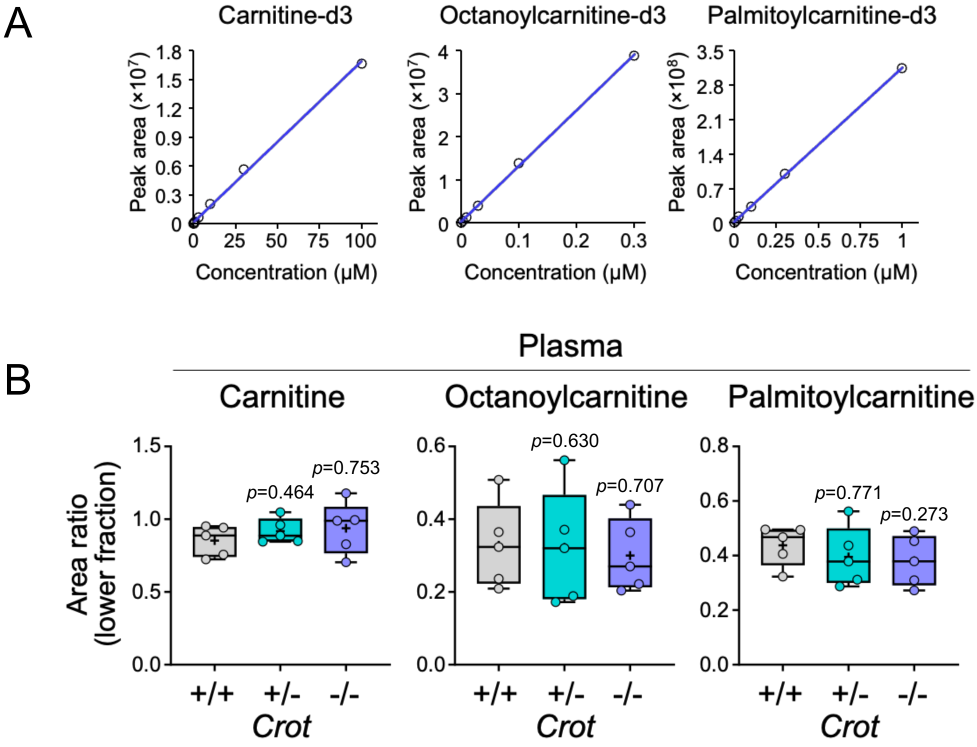


**Supplemental Figure 8**. L-carnitine and acyl-carnitine targeted MS2 data. A) Stable isotope-labeled (deuterium) standard curves. B) Box plots summarizing targeted mass spectrometry results for L-carnitine and acyl-carnitines measured in plasma in the indicated extraction fractions. Dunnett's multiple comparison test was performed (vs *Crot+/+,* n=5).
